# Prospective Study of the Performance of Parent-Collected Nasal and Saliva Swab Samples, Compared with Nurse-Collected Swab Samples, for the Molecular Detection of Respiratory Microorganisms

**DOI:** 10.1128/Spectrum.00164-21

**Published:** 2021-11-10

**Authors:** Claire A. Woodall, Hannah V. Thornton, Emma C. Anderson, Suzanne M. Ingle, Peter Muir, Barry Vipond, Denise Longhurst, John P. Leeming, Charles R. Beck, Alastair D. Hay

**Affiliations:** a Centre for Academic Primary Care, Population Health Sciences, Bristol Medical School, University of Bristol, Bristol, United Kingdom; b Centre for Academic Child Health, Population Health Sciences, Bristol Medical School, University of Bristol, Bristol, United Kingdom; c National Institute for Health Research, Health Protection Research Unit in Behavioral Science and Evaluation of Interventions, University of Bristol, Bristol, United Kingdom; d Centre for Academic Primary Care, University of Bristol, Bristol, United Kingdom; e Public Health England, Southwest Regional Laboratory, National Infection Service, Southmead Hospital, Bristol, United Kingdom; f Bristol Centre for Antimicrobial Research and Evaluation, Pathology Sciences, North Bristol NHS Trust, Bristol, United Kingdom; g Field Service, National Infection Service, Public Health England, Bristol, United Kingdom; Johns Hopkins Hospital

**Keywords:** respiratory tract infection, clinical methods, community-based, diagnostics, microbiology, molecular techniques, pediatric, parent collection, public health, self-collection

## Abstract

Respiratory tract infections (RTIs) are ubiquitous among children in the community. A prospective observational study was performed to evaluate the diagnostic performance and quality of at-home parent-collected (PC) nasal and saliva swab samples, compared to nurse-collected (NC) swab samples, from children with RTI symptoms. Children with RTI symptoms were swabbed at home on the same day by a parent and a nurse. We compared the performance of PC swab samples as the test with NC swab samples as the reference for the detection of respiratory pathogen gene targets by reverse transcriptase PCR, with quality assessment using a human gene. PC and NC paired nasal and saliva swab samples were collected from 91 and 92 children, respectively. Performance and interrater agreement (Cohen’s κ) of PC versus NC nasal swab samples for viruses combined showed sensitivity of 91.6% (95% confidence interval [CI], 85.47 to 95.73%) and κ of 0.84 (95% CI, 0.79 to 0.88), respectively; the respective values for bacteria combined were 91.4% (95% CI, 86.85 to 94.87%) and κ of 0.85 (95% CI, 0.80 to 0.89). In saliva samples, viral and bacterial sensitivities were lower at 69.0% (95% CI, 57.47 to 79.76%) and 78.1% (95% CI, 71.60 to 83.76%), as were κ values at 0.64 (95% CI, 0.53 to 0.72) and 0.70 (95% CI, 0.65 to 0.76), respectively. Quality assessment for human biological material (18S rRNA) indicated perfect interrater agreement. At-home PC nasal swab samples performed comparably to NC swab samples, whereas PC saliva swab samples lacked sensitivity for the detection of respiratory microbes.

**IMPORTANCE** RTIs are ubiquitous among children. Diagnosis involves a swab sample being taken by a health professional, which places a considerable burden on community health care systems, given the number of cases involved. The coronavirus disease 2019 (COVID-19) pandemic has seen an increase in the at-home self-collection of upper respiratory tract swab samples without the involvement of health professionals. It is advised that parents conduct or supervise swabbing of children. Surprisingly, few studies have addressed the quality of PC swab samples for subsequent identification of respiratory pathogens. We compared NC and PC nasal and saliva swab samples taken from the same child with RTI symptoms, for detection of respiratory pathogens. The PC nasal swab samples performed comparably to NC samples, whereas saliva swab samples lacked sensitivity for the detection of respiratory microbes. Collection of swab samples by parents would greatly reduce the burden on community nurses without reducing the effectiveness of diagnoses.

## INTRODUCTION

The coronavirus disease 2019 (COVID-19) pandemic has seen an increase in at-home self-collection of upper respiratory tract swab samples, which are then sent to the laboratory for clinical diagnostics (1, 2). The advantages of at-home self-collected swab samples over those collected by health care workers (HCWs) include convenience, reduced costs, and lower infection control risk to the HCWs (3).

Traditionally, respiratory tract infection (RTI) diagnosis is made by HCW collection of an invasive and uncomfortable nasopharyngeal (NP) swab sample, which is considered to have high sensitivity for viral detection (4). Reverse transcriptase PCR (RT-PCR) assays have increased the sensitivity of microbe detection from upper respiratory tract specimens, thus resulting in the collection of less-invasive, easy-to-obtain samples for RTI diagnosis, including nostril swab and saliva specimens (5, 6). However, assay accuracy is affected by several factors, including specimen type, swab tip material, and transport time to the laboratory (7–9). In addition, the person collecting the swab sample, for example, HCW, self, or parent, may impact assay performance.

Surprisingly, there are limited community-based studies demonstrating the performance of parent-collected (PC) swab samples obtained from children, compared to HCW-collected swab samples, for the detection of respiratory microbes (9–16). Many of those studies compared PC and HCW-collected swab samples from children but overlooked quality assessment measures (10–12, 15, 16). Those studies demonstrated the feasibility of PC swab samples, but further investigations are required to validate the diagnostic equivalence of parent versus HCW collection in a home environment, including a broad range of microbes and quality assessment measures. The aim of this community-based study was to compare the quality and performance of PC and nurse-collected (NC) nasal and saliva swab samples obtained from children with RTI symptoms for the detection of respiratory microbes.

## RESULTS

### Patients and samples.

**FIG 1:**
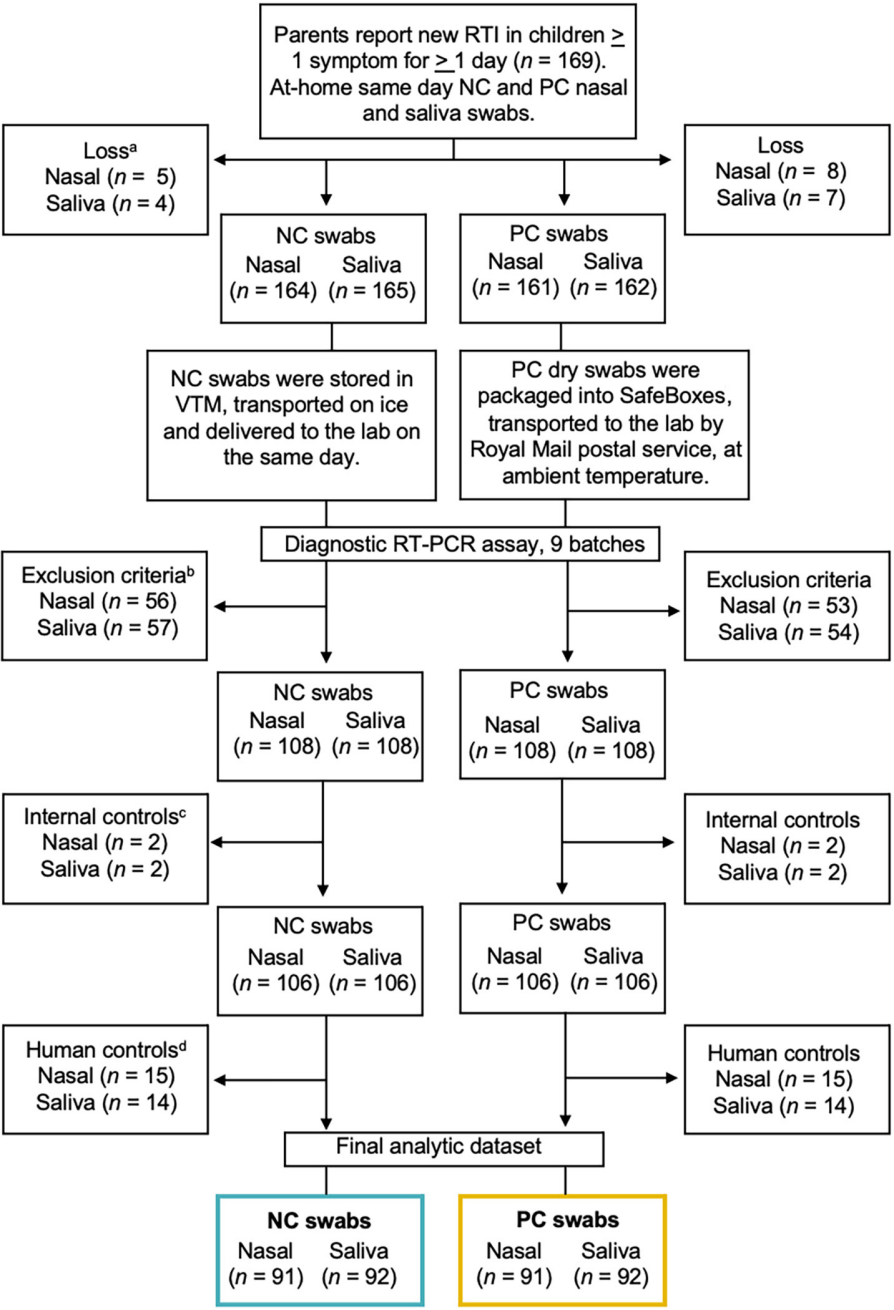
Data flow chart. a, Lost samples from the study were due to either a missed home visit, recovery of the child before the nurse visited, child refusal of the nurse and/or parent swabbing procedure, parental commitment hours, parents not being contactable, or form error. b, Exclusion criterion were applied to ensure analysis of a single swab test result from the first child in the family with RTI symptoms. c, Internal control genes were bacteriophage T4 and MS2. d, Human control genes were 18S rRNA and RNase P.

A total of 91 PC and NC nasal swab samples (*n *= 182) and 92 PC and NC saliva swab samples (*n *= 184) were analyzed for the detection of respiratory microbes (Fig. 1). Children analyzed in this secondary microbiology study had a median age of 2.6 years (interquartile range [IQR], 1.5 to 5 years). All swab samples were collected a mean of 6.4 days (95% confidence interval [CI], 5.8 to 6.9 days) after RTI symptom onset. All NC swab samples arrived at the laboratory on the same day, whereas PC swab samples arrived a mean of 2.7 days (95% CI, 2.33 to 3.06 days) after collection.

### Percent agreement.

To test whether parents could swab as effectively as nurses, we determined the percentage of paired swab samples that were RT-PCR positive for the detection of two control human gene targets, 18S rRNA and RNase P (Table 1). There was a difference in the detection of the RNase P gene between samples. Of paired nasal swab samples from 106 children, 97.16% (95% Cl, 94.1 to 100%) of PC swab samples, compared to 86.79% (95% Cl, 79.9 to 93.6%) of NC swab samples, were positive (*P *= 0.003). For the paired saliva swab samples, 88.67% (95% Cl, 82.3 to 95.1%) of PC swab samples, compared to 98.11% (95% Cl, 95.5 to 100%) of NC swab samples, were positive (*P *= 0.013). Thus, the RNase P gene demonstrated that PC nasal swab samples were better than NC nasal swab samples, whereas NC saliva swab samples were better than PC saliva swab samples. The complete agreement of PC and NC swab samples with regard to detection of the 18S rRNA gene indicates that parents can swab as effectively as nurses.

**TABLE 1:** Percentages of positive PC and NC nasal and saliva swab samples for the detection of human control genes

Sample type^*a*^	No. positive^*b*^/total no. (% [95% Cl])	
	NC samples	PC samples
Nasal swab samples	92/106 (86.79 [79.9–93.6])^*c*^	103/106 (97.16 [94.1–100])^*c*^
Saliva swab samples	104/106 (98.11 [95.5–100])^*d*^	94/106 (88.67 [82.3–95.1])^*d*^
Combined	196/212 (92.45 [87.2–97.6])	197/212 (92.92 [87.8–97.9])

a Paired samples were obtained from 106 children.

b Positive for detection of human control genes. The human control gene targets were 18S rRNA and RNase P; these allow the number of positive or negative test results to be compared between NC and PC swab samples. All test results were true-positive results (100%) for the detection of the 18S rRNA gene, indicating that PC and NC swab sample biological human material loads were the same, whereas there was less sensitivity for the detection of the RNase P gene. All negative-control data were removed from subsequent analysis of the identification of respiratory microbes.

c *P* < 0.01.

d *P* < 0.05.

For further analyses, however, paired samples in which either or both samples were negative for the detection of RNase P were excluded (Fig. 1). Such samples might have contained insufficient biological material, and a negative result for detection of specific pathogens could have been due to lack of material rather than an absence of pathogen, skewing the analysis of pathogen detection.

### Quality assessment.

Interrater reliability and sensitivity were used to assess the performance of the PC and NC swab samples using detection of the human 18S rRNA gene, an indication that the swab contained biological material. The PC nasal and saliva sample 18S rRNA gene results showed 100% sensitivity and specificity and a κ value of 1, indicating perfect agreement with the NC swab results (Table 2).

**TABLE 2:** Results for PC and NC nasal and saliva swab pairs for the detection of respiratory microbes and a human control gene (18S rRNA)

Microbe	No.				Sensitivity (% [95% Cl])^*b*^	Specificity (% [95%Cl])^*b*^	Prevalence (% [95% Cl])^*b*^	κ (95%Cl)^*b*^
	TP^*a*^	TN	FP	FN				
Nasal microbes								
M. catarrhalis	79	9	2	1	98.75 (93.23–99.97)	81.82 (48.22–97.72)	87.91 (79.40–93.81)	0.84 (0.66–1)
Rhinovirus 2	44	34	10	3	93.62 (82.46–98.66)	77.27 (62.16–88.53)	51.65 (40.93–62.26)	0.71 (0.57–0.86)
Rhinovirus	43	38	7	3	93.48 (82.10–98.63)	84.44 (70.54–93.51)	50.55 (39.86–61.20)	0.78 (0.65–0.91)
S. pneumoniae	45	39	6	1	97.83 (88.47–99.94)	86.67 (73.21–94.95)	50.55 (39.86–61.20)	0.85 (0.74–0.95)
H. influenzae	42	37	10	2	95.45 (84.53–99.44)	78.72 (64.34–89.30)	48.35 (37.74–59.07)	0.74 (0.60–0.87)
Coagulase-negative Staphylococcus species	14	54	15	8	63.64 (40.66–82.80)	78.26 (66.69–87.29)	24.18 (15.81–34.28)	0.38 (0.17–0.58)
Influenza B quadrivalent	5	80	3	3	62.50 (24.49–91.48)	96.39 (89.80–99.25)	8.79 (3.87–16.59)	0.59 (0.29–0.89)
S. pyogenes	7	79	4	1	87.50 (47.35–99.68)	95.18 (88.12–98.67)	8.79 (3.87–16.59)	0.71 (0.47–0.95)
Enterovirus	7	84	0	0	100 (59.04–100)	100 (95.70–100)	7.69 (3.15–15.21)	1
S. aureus	4	80	4	3	57.14 (18.41–90.10)	95.24 (88.25–98.69)	7.69 (3.15–15.21)	0.49 (0.10–1)
Coronavirus NL63	5	86	0	0	100 (47.82–100)	100 (95.80–100)	5.49 (1.81–12.36)	1
Bocavirus	3	86	2	0	100 (29.24–100)	97.73 (92.03–99.72)	3.3 (0.69–9.33)	0.74 (0.39–1)
Parainfluenza 2 and 3	2	88	0	1	66.67 (19.43–99.16)	100 (95.89–100)	3.3 (0.69–9.33)	0.79 (0.43–1)
Adenovirus C	2	86	3	0	100 (15.81–100)	96.63 (90.46–99.30)	2.2 (0.27–7.71)	0.74 (0.39–1)
Adenovirus B	2	87	2	0	100 (15.81–100)	97.75 (92.12–99.73)	2.2 (0.27–7.71)	0.66 (0.21–1)
Influenza B (Bruges)	2	89	0	0	100 (15.81–100)	100 (95.94–100)	2.2 (0.27–7.71)	1
Parainfluenza 3	2	88	1	0	100 (15.81–100)	98.88 (93.90–99.97)	2.2 (0.27–7.71)	0.79 (0.43–1)
* * Mycoplasma pneumoniae	0	89	0	2	0 (0–84.19)	100 (95.94–100)	2.2 (0.27–7.71)	0
Enterovirus D68	0	90	0	1	0 (0–97.50)	100 (95.98–100)	1.1 (0.03–5.97)	0
Metapneumovirus	1	89	1	0	100 (2.50–100)	98.89 (93.96–99.97)	1.1 (0.03–5.97)	0.66 (0.04–1)
Parainfluenza 4	1	90	0	0	100 (2.50–100)	100 (95.98–100)	1.1 (0.03–5.97)	1
Parechovirus	1	89	1	0	100 (2.50–100)	98.89 (93.96–99.97)	1.1 (0.03–5.97)	0.66 (0.04–1)
N. meningitidis	1	90	0	0	100 (2.50–100)	100 (95.98–100)	1.1 (0.03–5.97)	1
Met resistance	1	88	2	0	100 (2.50–100)	97.78 (92.20–99.73)	1.1 (0.03–5.97)	0.49 (0.17–0.82)
Viruses^*c*^ combined	120	1,204	30	11	91.60 (85.47–95.73)	97.57 (96.55–98.35)	9.60 (8.09–11.28)	0.84 (0.79–0.88)
Bacteria^*d*^ combined	193	565	43	18	91.47 (86.85–94.87)	92.93 (90.59–94.83)	25.76 (22.80–28.90)	0.85 (0.80–0.89)
Human control gene, 18S rRNA	91	0	0	0	100	100	100	1
Salivary microbes								
Coagulase-negative Staphylococcus species	69	8	10	5	93.24 (84.93–97.77)	44.44 (21.53–69.24)	80.43 (70.85–87.97)	0.41 (0.18–0.66)
H. influenzae	27	39	13	13	67.50 (50.87–81.43)	75 (61.05–85.97)	43.48 (33.17–54.22)	0.43 (0.24–0.61)
S. pneumoniae	23	45	11	12	65.71 (47.79–80.87)	80.36 (67.57–89.77)	38.46 (28.45–49.25)	0.46 (0.28–0.65)
M. catarrhalis	25	45	14	8	75.76 (57.74–88.91)	76.27 (63.41–86.38)	35.87 (26.13–46.54)	0.5 (0.32–0.68)
Rhinovirus 2	16	62	8	6	72.73 (49.78–89.27)	88.57 (78.72–94.93)	23.91 (15.63–33.94)	0.59 (0.40–0.79)
Rhinovirus	19	60	11	2	90.48 (69.62–98.83)	84.51 (73.97–92.00)	22.83 (14.72–32.75)	0.65 (0.48–0.82)
Bocavirus	1	85	2	4	20.00 (0.51–71.64)	97.70 (91.94–99.72)	5.43 (1.79–12.23)	0.22 (0.19–0.62)
Enterovirus	4	87	1	0	100 (39.76–100)	98.86 (93.83–99.97)	4.35 (1.20–10.76)	0.88 (0.66–1)
Parechovirus	2	87	1	2	50 (6.76–93.24)	98.86 (93.83–99.97)	4.35 (1.20–10.76)	0.55 (0.11–1)
Influenza B quadrivalent	0	87	2	3	0 (0–70.76)	97.75 (92.12–99.73)	3.26 (0.68–9.23)	0
Human parainfluenza virus 3 and 2	2	89	0	1	66.67 (9.43–99.16)	100 (95.94–100)	3.26 (0.68–9.23)	0.49 (0.11–1)
Coronavirus NL63	2	88	1	1	66.6 (79.43–99.16)	98.88 (93.90–99.97)	3.26 (0.68–9.23)	0.65 (0.21–1)
S. pyogenes	3	89	0	0	100 (29.24–100)	100 (95.94–100)	3.26 (0.68–9.23)	1
Adenovirus 2	1	89	1	1	50 (1.26–98.74)	98.89 (93.96–99.97)	2.17 (0.26–7.63)	0.49 (0.12–1)
Adenovirus	1	87	3	1	50 (1.26–98.74)	96.67 (90.57–99.31)	2.17 (0.26–7.63)	0.31 (0.18–0.81)
Met resistance	2	89	1	0	100 (15.81–100)	98.89 (93.96–99.97)	2.17 (0.26–7.63)	0.79 (0.40–1)
N. meningitidis	0	90	0	2	0 (0–84.19)	100 (95.98–100)	2.17 (0.26–7.63)	0
Influenza B (Bruges)	0	91	0	1	0 (0–97.50)	100 (96.03–100)	1.09 (0.03–5.91)	0
Human metapneumovirus	1	91	0	0	100 (2.50–100)	100 (96.03–100)	1.09 (0.03–5.91)	1
Human parainfluenza virus 3	0	90	1	0	100 (2.50–100)	98.90 (94.03–99.9)	1.09 (0.03–5.91)	0
M. pneumoniae	0	91	0	1	0 (0–97.50)	100 (96.03–100)	1.09 (0.03–5.91)	0
* * Bordetella pertussis	0	91	0	1	0 (0–97.50)	100 (96.03–100)	1.09 (0.03–5.91)	0
F. necrophorum	1	91	0	0	100 (2.50–100)	100 (96.03–100)	1.09 (0.03–5.91)	1
Viruses combined	49	1,093	31	22	69.01 (57.47–79.76)	97.24 (96.11–98.12)	5.94 (4.67–7.44)	0.64 (0.53–0.72)
Bacteria combined	150	678	49	42	78.12 (71.60–83.76)	93.26 (91.19–94.97)	20.89 (18.31–23.67)	0.70 (0.65–0.76)
Human control gene, 18S rRNA	92	0	0	0	100	100	100	1

a TP, true-positive result (both PC and NC samples were positive for detection of the microbe); TN, true-negative result (both PC and NC samples were negative for detection of the microbe); FN, false-negative result (NC sample was positive and PC sample was negative); FP, false-positive result (NC sample was negative and PC sample was positive).

b NC samples were considered the reference standard for calculating the sensitivity, specificity, prevalence, and κ value.

c Viruses combined refers to the number of tests with at least one positive RT-PCR result. A total of 29 viral gene targets were analyzed in pair-matched swab samples.

d Bacteria combined refers to the number of tests with at least one positive RT-PCR result. A total of 15 bacterial gene targets were analyzed in pair-matched swab samples.

**FIG 2:**
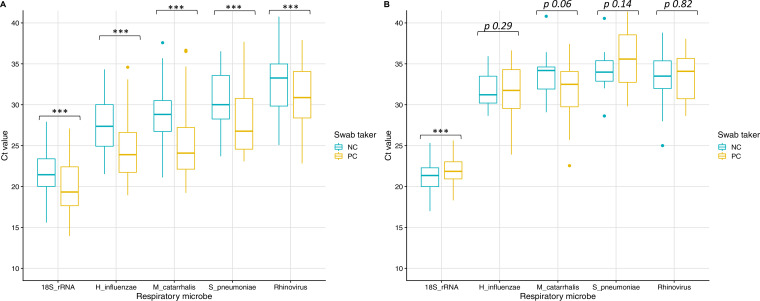
*C_T_* values for the most prevalent respiratory microbes and the human control gene (18S rRNA) in PC and NC nasal (A) and saliva (B) swab samples. The human rhinovirus and rhinovirus 2 *C_T_* values were combined. Nasal PC and NC human gene and microbe gene targets demonstrated significant differences (*P* < 0.001) whereas saliva PC and NC human gene target demonstrated a significant difference (*P* < 0.001), all other values can be found in the results section.

To assess the amounts of human biological material collected on the PC and NC swab samples, the 18S rRNA cycle threshold (*C_T_*) values were analyzed. The *C_T_* value for the detection of 18S rRNA was significantly (*P *= 1.1 × 10^−6^) lower (higher expression) for PC nasal swab samples (19.32 [95% CI, 18.66 to 19.99]) than for NC swab samples (21.44 [95% CI, 20.89 to 21.98]) (Fig. 2A). For paired saliva swab samples, the mean *C_T_* value was significantly (*P *= 0.001) higher (lower expression) for PC swab samples (21.85 [95% CI, 21.53 to 22.17]) than for NC swab samples (21.35 [95% CI, 20.99 to 21.69]) (Fig. 2B). This indicates that PC nasal swab samples contained more biological material than did NC nasal swab samples but NC saliva swab samples contained more biological material than did PC saliva swab samples.

A comparison plot and agreement by Bland-Altman analysis indicated that, regardless of the site (nasal swab samples versus saliva swab samples) or who collected the specimen (PC swab samples versus NC swab samples), the majority of 18S rRNA *C_T_* values were within the limits of agreement (see Fig. S2 in the supplemental material). In this study, the mean RNase P gene *C_T_* values were higher than the mean 18S rRNA *C_T_* values, indicating lower expression levels or lower assay sensitivity. Overall, the RNase P gene results were less reliable and were not trusted as a suitable control for this study (Fig. 1).

### PC swab sample performance.

To compare the performance of PC swab samples with NC swab samples for the detection of microbes, only pairs of PC and NC swab samples in which both were positive for both human control genes were considered (Fig. 1). In total, there were 57% and 43% RT-PCR assay-positive results for at least one microbe among PC nasal and saliva swab samples, respectively. Among NC nasal and saliva swab samples, there were 56% and 44% positive results, respectively. A full breakdown of the detection of individual microbes is presented in Table S2 in the supplemental material.

When the performance of PC nasal swab samples was compared with that of the reference NC nasal swab samples, the sensitivity and specificity for the detection of viruses were 91.60% (95% CI, 85.47 to 95.73%) and 97.57% (95% CI, 96.55 to 98.35%), respectively; for bacteria, the sensitivity and specificity of the PC nasal swab samples were 91.47% (95% CI, 86.85 to 94.87%) and 92.93% (95% CI, 90.59 to 94.83%), respectively (Table 2). The nasal microbes demonstrating 100% sensitivity and specificity in PC swab samples, compared to NC swab samples, were enterovirus, coronavirus NL63, influenza B (Bruges), parainfluenza 4, and Neisseria meningitidis (Table 2).

For saliva swab samples, the sensitivity and specificity of PC swab samples, compared to NC swab samples, for detection of viruses were 69.01% (95% CI, 57.47 to 79.76%) and 97.24% (95% CI, 96.11 to 98.12%), respectively; for bacteria, they were 78.12% (95% CI, 71.60 to 83.76%) and 93.26% (95% CI, 91.19 to 94.97%), respectively (Table 2). Salivary microbes metapneumovirus, Streptococcus pyogenes, and Fusobacterium necrophorum showed 100% sensitivity and specificity in PC swab samples, compared to NC swab samples (Table 2).

### Interrater reliability.

Overall, the interrater reliability between PC and NC swab samples for detection of nasal viruses and bacteria showed excellent agreement, with κ values of 0.84 (95% CI, 0.79 to 0.88) and 0.85 (95% CI, 0.89 to 0.90), respectively (Table 2). Perfect agreement of PC nasal swab samples with NC swab samples was shown for the detection of enterovirus, coronavirus NL63, influenza B (Bruges), parainfluenza 4, and N. meningitidis (Table 2).

The interrater reliability between PC and NC swab samples for detection of salivary viruses and bacteria showed substantial agreement, with κ values of 0.64 (95% CI, 0.53 to 0.72) and 0.70 (95% CI, 0.65 to 0.76), respectively (Table 1). Perfect agreement was observed for detection of metapneumovirus, S. pyogenes, and F. necrophorum (Table 2).

### Respiratory microbe prevalence.

The most prevalent microbes detected in nasal swab samples were Moraxella catarrhalis (87.91% [95% CI, 79.40 to 93.81%]) and rhinovirus 2 (51.65% [95% CI, 40.93 to 62.26%]), whereas those in saliva swab samples were coagulase-negative staphylococci (80.43% [95% CI, 70.85 to 87.97]) and rhinovirus 2 (23.91% [95% CI, 15.62 to 33.94]) (Table 2).

### Respiratory microbe *C_T_* values.

The *C_T_* values for detection of the most prevalent viruses (rhinovirus and rhinovirus 2 combined data), bacterial pathobionts (Haemophilus influenzae, M. catarrhalis, and Streptococcus pneumoniae), and the human control gene (18S rRNA) in nasal swab samples (Fig. 2A) and saliva swab samples were plotted (Fig. 2B). In nasal swab samples, there were significant differences in the *C_T_* values between PC and NC swab samples for the detection of H. influenzae (*P *= 9.3 × 10^−7^), M. catarrhalis (*P *= 6.6 × 10^−12^), S. pneumoniae (*P *= 7 × 10^−6^), and rhinoviruses (*P *= 2.5 × 10^−6^) (Fig. 2A). PC and NC swab sample microbial *C_T_* values were within the IQRs. This demonstrates that PC nasal swab samples, compared to NC nasal swab samples, had a larger amount of microbes. Among saliva swab samples, there was no significant difference in the *C_T_* values between PC and NC swab samples for detection of H. influenzae (*P *= 0.29), M. catarrhalis (*P *= 0.06), S. pneumoniae (*P *= 0.14), and rhinoviruses (*P *= 0.82) (Fig. 2B). PC and NC swab sample microbial *C_T_* values were within the IQRs. This indicates that PC and NC saliva swab samples have very similar amounts of microbes.

## DISCUSSION

We have demonstrated that PC nasal swab samples collected at home from children displaying RTI symptoms are comparable to NC swab samples for the detection of respiratory viruses and bacteria. Although PC saliva samples demonstrated high levels of specificity, they were insufficiently sensitive to substitute for NC samples.

Reassuringly, expected respiratory pathobionts and viruses were detected in both nasal and saliva swab samples. The majority of the nasal microbes fell within the TaqMan Array Card (TAC) clinical validation sensitivity range of 89.1% to 100% (17). Consistent with our findings, nasal colonization by S. pneumoniae has been positively associated with the presence of H. influenzae, M. catarrhalis, rhinoviruses, and enteroviruses and negatively associated with the presence of Staphylococcus aureus (18). Our study indicated that children with RTI symptoms had a high prevalence of rhinovirus, whereas enterovirus, coronavirus NL63, influenza B, and parainfluenza 4 were less prevalent, which is consistent with other studies (9–13, 16, 19). Zoch-Lesniak and colleagues tested three pathobionts, i.e., M. catarrhalis, S. pneumoniae, and H. influenzae, which showed positive agreement ranging from 64 to 77% (14). In our study, we showed that these three pathobionts demonstrated high sensitivity ranging from 95.45 to 98.75% in nasal swab samples, and we also tested for a number of other microbes, some of which, such as coronavirus NL63, showed perfect agreement in PC swab samples.

Increasingly, saliva has been used for the clinical detection of respiratory pathogens during an RTI (20, 21). In children with RTI symptoms, the collection of an upper respiratory tract swab sample is particularly challenging, whereas collecting saliva into a sponge swab from the base of the mouth was the least invasive sampling option. In this study, we found that saliva was less sensitive for the detection of respiratory microbes. Possible reasons for finding a lower number of microbes in saliva, compared to the nasal cavity, include the following: (i) the function of saliva is to wash away food and microbial debris, (ii) the feasibility study reported that several parents described the mouth swabs as being more difficult to use than the nasal swabs, (iii) the collection of saliva involved the sponge swab being placed in the mouth for a certain time, which might have been challenging for children to tolerate, and (iv) some of the youngest infants and toddlers reportedly disliked the saliva swabs (22). Previous studies on adult saliva samples for the detection of severe acute respiratory syndrome coronavirus 2 (SARS-CoV-2) and other viruses demonstrated variable results, which should be interpreted with caution (5, 23).

Results for detection of the 18S rRNA gene in PC and NC samples were in perfect agreement. However, there were differences in the mean *C_T_* values for PC and NC samples, indicating a difference in the amounts of biological material collected (Fig. 2). This was mirrored in the observed *C_T_* values for the detection of respiratory microbes. RT-PCR-based assays have limitations and are highly sensitive, highlighting *C_T_* value variability when measuring human cell loads harvested by swabbing different upper respiratory tract sample sites or comparing collection methods (24). We found that PC nasal swab samples contained more biological material and respiratory microbes, compared to NC swab samples. We speculate that parents’ experience with their children allowed them to better predict their tolerance for nostril swabbing and that nurses might have been more motivated not to upset the children. A previous study evaluated the efficiency of midturbinate swab samples for the detection of influenza virus among 203 children and demonstrated that parents were the preferred swabbers, compared to HCWs (19). In our study, NC saliva swab samples contained more biological material and equivalent respiratory microbes, compared to PC samples. This suggests that a sponge swab placed in the mouth to soak up saliva for a defined time period yields consistent results, compared to the physical pressure required to harvest a swabbed specimen from the nostril. Overall, considering the different methods for the swab collection of a specimen, the biological loads, and detected microbes, we found that PC nostril swab samples are comparable to, if not better than, NC swab samples, whereas PC saliva swab samples are less comparable to NC samples.

To our knowledge, this is the first study to compare PC and NC nasal and saliva samples in young children with RTI symptoms, taken at home, for the identification of a comprehensive range of respiratory viruses and bacteria, including a quality assessment measure. However, there are several limitations to this study. A total of 183 children were recruited and swabbed by their parent and a nurse, yielding both nasal and saliva swab specimens (366 pair-matched swab samples for molecular diagnostics). This suggests that there was adequate sampling of the community to ensure performance validity between PC and NC swab samples, compared to other community-based studies in which 33 to 234 children were recruited and swabbed for the detection of respiratory pathogens (9–16, 19, 25).

To assess performance, we used the NC swab sample RT-PCR results as the reference standard against which the performance of PC swab samples was compared. A true reference standard is defined as “the best available method for establishing the presence or absence of the target condition” (26). Therefore, we considered the community nurse the most qualified individual for collection of an upper respiratory tract swab sample from a child with RTI symptoms at home.

In our study, the RNase P gene generated a number of false-positive and false-negative results and, with a low expression level, it was not used as a control. There is no standard human housekeeping gene for performance assessment measures; therefore, judgement is required in each study. The endogenous human retrovirus gene (ERV3) was used previously to assess biological loads between nasal and NP swab samples, comparing swab collection by a parent and a HCW (14). Our results demonstrated perfect agreement for PC swabbed human cells, compared to NC swab samples, for the 18S rRNA gene.

In this study, two measures were used to compare the performance of PC and NC nasal and saliva swab samples. Cohen’s κ value is widely used but involves subjective interpretation. This interpretation, by Landis and Koch (27), categorizes κ values into different levels of agreement and is widely used. When the κ values are high (1.00 to 0.81 [excellent]), the interpretation is relatively straightforward, whereas lower values are more subjective and could be unintentionally misleading (28). Other studies have recommended more than one approach for performance comparisons (28). We used percent agreement (based on the binomial RT-PCR result, which is not subject to conjecture), which supports the κ value interpretation demonstrating that PC nasal swab samples and NC nasal swab samples were comparable for most detected microbes, whereas PC saliva samples were inadequately sensitive to substitute for NC swab samples.

Given the widespread use of nasal sampling in the COVID-19 pandemic, 384 million lateral-flow at-home self-test kits are already being used throughout the United Kingdom, advocating the ease of nostril swabbing (29). COVID-19 self-test kits are suitable for parent collection of nasal swab samples from children 6 to 12 years of age. The requirement for a low-cost, easy-to-collect, point-of-care kit to use at home to detect RTIs should be considered globally and further explored to assess the performance of teenage self-collected swab samples. In conclusion, our study shows that at-home PC nasal swab samples from young children with RTI symptoms, rather than saliva swab samples, appear to be satisfactory for the molecular detection of respiratory microbes.

## MATERIALS AND METHODS

### Study population.

Recruitment, data collection, and demographic information for children were described previously (22). In brief, children with a median age of 4 years (IQR, 2 to 8 years) were recruited before the COVID-19 pandemic; most were white (Caucasian), with no underlying health conditions (22). Microbiological analyses were restricted to the first RTI episode per child and were performed with pair-matched PC and NC nasal and saliva swab samples (Fig. 1). The Southwest Frenchay Bristol Research Ethics Committee approved the study, including the consent processes and participant recruitment (reference number 15/SW/0264).

### Upper respiratory tract specimen collection.

Parents were given written instructions on how to collect effective nasal swab and saliva swab samples (see Fig. S1 in the supplemental material). The swab home kits were provided by the nurses on the day they visited the family home, to allow parents ample time to read the swab collection instructions. PC nasal swab samples were collected with a sterile dry PurFlock nylon swab (Medical Wire and Equipment, UK), placed just inside the nostril entrance and rotated three times. Saliva sponge Oracol swabs (Malvern Medical Developments, UK) were placed in the mouth under the tongue for at least 1 min, until approximately 1 ml of crevicular fluid soaked into the swab. PC samples were packaged into a first-class Royal Mail SafeBox and posted to the laboratory at ambient temperature. Nurses swabbed the children immediately after the parents had collected swab samples. The NC nasal swab was immersed in a vial containing 1 ml viral transport medium (VTM) and then both of the NC swab samples were placed on ice and delivered to the laboratory the same day.

### Laboratory processing and nucleic acid extraction.

Upon laboratory receipt, nasal swab samples and saliva samples were stored at –80°C until required. Batches of samples for extraction were allowed to thaw at room temperature. The volume of saliva in the collection tubes was estimated. If necessary, low-volume specimens (<200 μl) were diluted with 200 μl phosphate-buffered saline. Dry nasal swab samples were prepared by adding 1 ml VTM from sterile Copan tubes and vortex-mixing briefly to agitate the swab. Then, 100 μl of each sample was extracted using the QIAsymphony DSP virus pathogen minikit (Qiagen) and the 60-μl elution protocol, including an internal process control containing bacteriophages T4 and MS2.

### TaqMan low-density array.

To detect respiratory microbes (29 viruses and 13 bacteria), 2 exogenous extraction controls (T4 and MS2 bacteriophages), and 2 endogenous human control genes (18S rRNA and RNase P genes), a 42-microbe TAC (Applied Biosystems, Foster City, CA, USA) was used (see Table S1). The TAC was clinically validated at Southmead Hospital (Bristol, UK) (17). Upon completion of the amplification reactions, fluorescence traces were inspected and analyzed for sigmoidal curves. Baselines and thresholds were set automatically using the software algorithms or, where necessary, by manual adjustment to avoid fluorescence noise. A *C_T_* value of <38 for any gene target was reported as a positive result. Samples were run in 9 batches and were amplified and analyzed using a Life Technologies Custom TaqMan low-density array system on an Applied Biosystems Life Technologies ViiA-7 real-time PCR system, as described elsewhere (30).

### Statistical analysis.

The performance of PC samples relative to NC samples was assessed by calculating sensitivity, specificity, and interrater agreement using Cohen’s κ (31). The values of Cohen's k coefficients were interpreted according to the method of Landis and Koch, as follows: 1.00 to 0.81, excellent; 0.80 to 0.61, good (substantial); 0.60 to 0.41, moderate; 0.40 to 0.21, weak; 0.20 to 0.00, negligible agreement (27).

PC swab samples were considered the test, and NC swab samples were the reference standard. Positive test results for microbes by both methods were defined as true-positive results. A positive PC sample test result with a negative NC sample test result was defined as a false-positive result, whereas a positive NC sample test result with a negative PC sample test result was defined as a false-negative result. Prevalence was calculated as the number of tests positive for a microbe (true-positive results) divided by the total number of tests for the microbe. McNemar’s test (32) was used to assess differences between the percentages of positive and negative RT-PCR test results for paired PC and NC swab samples. The Wilcoxon signed-rank test (33) was used to determine differences between PC and NC sample gene target mean *C_T_* values. A Bland-Altman plot (34) was created in Microsoft Excel (version 16.42) to demonstrate the limits of agreement between PC sample and NC sample *C_T_* values for the 18S rRNA control gene.

MedCalc and GraphPad QuickCals software were used to calculate sensitivity, specificity, prevalence, and κ. To demonstrate equivalence between PC sample and NC sample *C_T_* value comparison plots, boxplots and statistics were performed in R via RStudio (version 3.3.2).
